# IDSEM, an invoices database of the Spanish electricity market

**DOI:** 10.1038/s41597-022-01885-3

**Published:** 2022-12-26

**Authors:** Javier Sánchez, Agustín Salgado, Alejandro García, Nelson Monzón

**Affiliations:** Centro de Tecnologías de la Imagen (CTIM), Computer Science Department, University of Las Palmas de Gran Canaria (ULPGC), Las Palmas de Gran Canaria, 35017 Spain

**Keywords:** Power distribution, Computer science, Scientific data, Energy modelling

## Abstract

This article describes a new database of electricity bills related to energy consumption in Spanish households. The dataset includes individual invoices containing information about the consumption and billing of each supply point. These documents include additional data about the customer, the contract, and the electricity company. We propose a pipeline for the creation of bill contents through a simulation process based on regulations and statistics from official bodies and electricity companies. This makes it possible to generate many documents with synthetic data. The simulation is based on 86 different labels, which are necessary to create realistic invoices. The dataset has 75 000 documents in PDF format with their corresponding labels in JSON files. It is useful for training machine learning algorithms and, in particular, for developing methods to automatically extract information from the bills. It is also interesting to design new algorithms for analyzing the behavior of electricity markets from different perspectives.

## Background & Summary

The electricity market in Spain has undergone many changes during the last two decades. It has adopted the policies of the European Union (EU), whose main objective is to establish common rules for all countries, promoting competition and transparency. Additionally, the production model is changing towards a more sustainable system based on renewable energies. Nevertheless, there has been an increase in electricity demand and production costs, which translates into higher household consumption and billing.

Understanding and analyzing the energy data is difficult due to the number of actors that intervene in its production, distribution, and commercialization, and also to the complexity of the pricing system. In the case of the Spanish electricity market, more than four hundred marketers operate in autonomous regions, with a few companies providing their services nationwide. Fortunately, the market is regulated, and the contents of invoices are similar regardless of the marketer that issues them.

Extracting information from invoices is an efficient way to analyze the behavior of the market as they reflect many aspects of the system. It would be desirable to have a large dataset of bills, although it is difficult to obtain many documents due to data protection laws. In addition, it is also laborious to obtain, anonymize and annotate many of them correctly. One way to get around this problem is to simulate their contents.

In this work, we design a new database of randomly generated invoices. Based on a set of bills from several marketers, we create a large set of documents with simulated data. The bills include information about the customer, the marketer, the distributor, the electricity consumption, and the billing data. We have used actual information and statistics about the market from official bodies, such as the *Comisión Nacional de los Mercados y la Competencia* (CNMC) and *Red Eléctrica Española* (REE).

The dataset has 75 000 invoices in PDF format organized in two directories: 30 000 in the training directory and 45 000 in the test directory. This organization is especially useful for training machine learning algorithms. Each invoice is defined by 86 different labels, which are necessary to model all the concepts that are included in electricity invoices. The labels of each invoice are written in JSON files.

There exist several datasets about energy consumption and billing. For example, a dataset of a house in Canada^1^ includes measurements from the electricity meter and billing information. Similar information is collected in other countries, like in Korea^2^, with the consumption of 22 houses, or in UK^3^ from 255 households. An electricity demand dataset of individual appliances in Germany^4^ contains recordings of 15 houses over a period of 3.5 years. Also in Germany, a dataset^5^ was created from 38 single-family houses with residential electricity information and heat pump load profiles. Another dataset^6^ includes 96 days of appliance consumption from one household in Portugal, and a dataset in Uruguay^7^ contains consumption and billing data conformed by the total household consumption, electric water heater consumption, and by-appliance electricity consumption. A dataset about electricity usage in US^8^ contains hourly demand data from balancing authorities. A work on time series of energy consumption^9^ provides data for heat, cold, mechanical energy, information and communication, and light in high spatial and temporal resolution.

The main difference with respect to our dataset is that ours is composed of individual invoices, which is a more general source of information. Most of the previous works include tabular information from a few houses. The SROIE^10^ and CORD^11^ datasets are similar to our work, containing one thousand scanned receipts each, although not related to the electricity market. In these cases, the receipts were manually annotated and the number of labels was small.

The main goal that motivated the creation of the dataset is to facilitate the study of new machine-learning algorithms to extract information from invoices. Information extraction from documents is an important task in the field of natural language processing. Given that this type of invoice reflects the reality of the electricity market, our dataset can contribute to the development of applications that allow a bottom-up analysis of energy consumption and billing, having special interests from an economic and social point of view.

For example, a web application may allow customers to upload their bills, visualize their contents, and compare them with the information of other customers. The database can be used to test the application with many bills before it goes into production. It can also be used for testing similar applications, such as a marketplace where customers may compare some offers from different companies using their own data.

## Methods

In this section, we describe the typical contents of electricity invoices, the design of the database with the main steps of the pipeline, and the process to produce the data of each invoice, explaining how we simulate the data and the sources of information.

### Content of electricity invoices

The content of electricity invoices is regulated in the *Boletín Oficial del Estado* (BOE)^12^. This regulation contributes to higher transparency for the customer, however, it contains so many details that it is difficult to understand all the concepts and their relationships in general. Invoices hold information about the customer, the marketer, the electricity consumption and prices, and supplementary information, such as the evolution of the total amounts paid by the customer during the last months, or the origin of the electricity, whether it is from renewable or coal-based production systems.

**Fig. 1:**
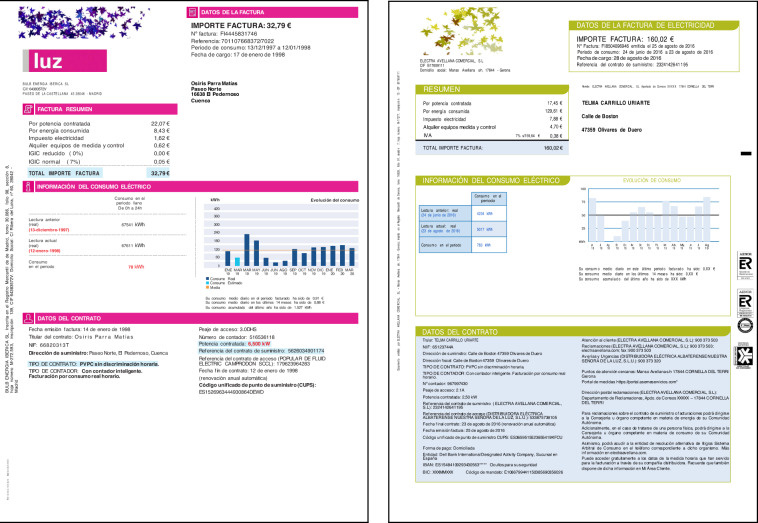
Layout examples of invoices from several marketers. These images show the first page of invoices from several companies. An arbitrary image has replaced the logo of companies.

**Fig. 2:**
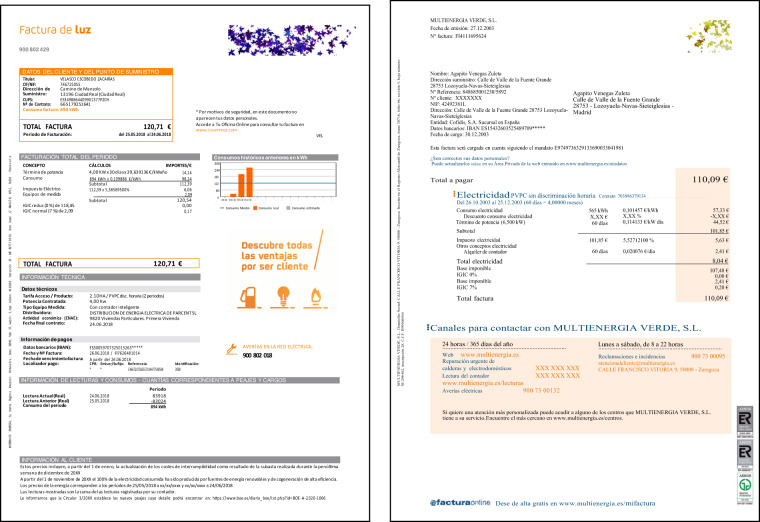
More examples of invoices from several marketers. These images show the first page of invoices from several companies. An arbitrary image has replaced the logo of companies.

Most marketers include several pages in each invoice: the first page typically summarizes the billing data and customer information, and the second and subsequent pages introduce detailed information about the consumption, the contract, and the breakdown of the invoice. The layout of bills, on the other hand, is different for each company. Elements, such as tables, field-value texts, or graphics, are organized arbitrarily, and the information is usually grouped in boxes, sometimes with additional information for making it more comprehensible. Figures 1, 2 show the layout of four invoices.

**Fig. 3:**
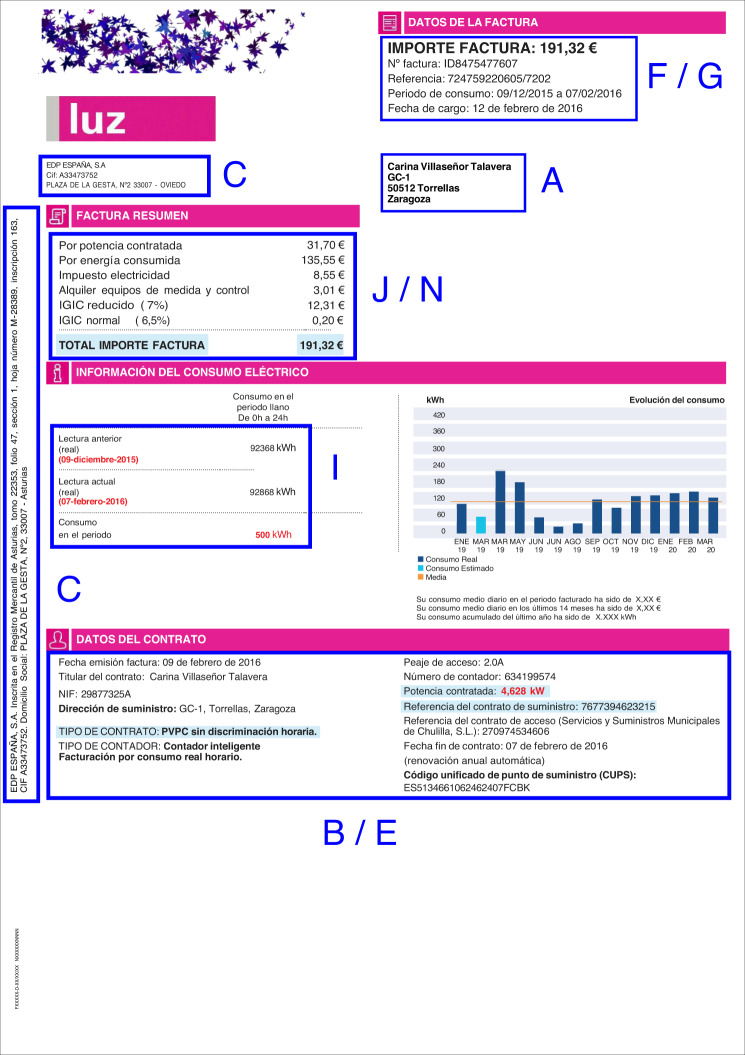
Layout of an electricity invoice. Blue boxes group the main contents of the document, organized by the marketer information (C), customer data (A), bill amounts (J,N), electricity consumption (I), contract information (F,G), and the breakdown (B,E).

**Fig. 4:**
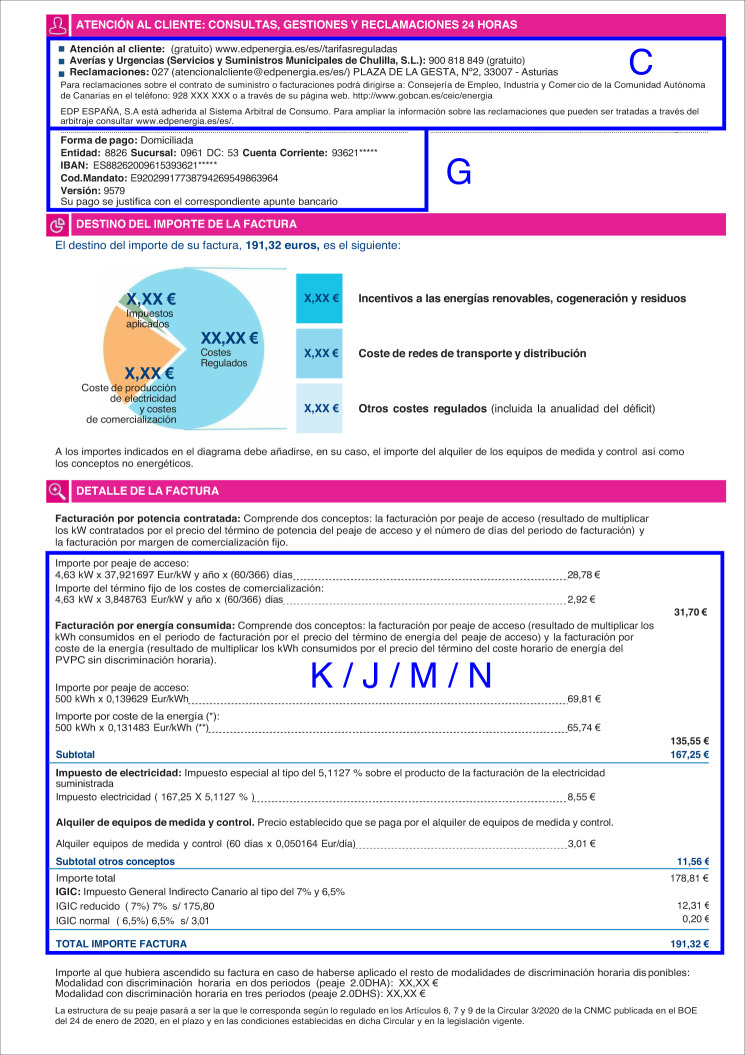
Layout of an electricity invoice. A second page usually includes more details about the invoice. In this example, blue boxes are organized by the marketer information (C), the bank account data (G), bill amounts (J,N), and other breakdown concepts (K, M).

In the case of the document shown in Figs. 3, 4, the information is organized in several groupings: the marketer address and its tax identification code are situated below the company logo (group C); on the right side, there is general information about the bill, such as the invoice number, the issue date, or the billing period (groups F and G); the customer data are placed below this group (group A); a summary of the bill amounts is situated at the beginning of the document body (groups J and N) and a summary of the energy consumption is given below (group I); finally, at the bottom of the page, there is information about the contract (groups B and E). There are also some graphics with information about the evolution of consumption.

On the next page, we find information about the contract, a breakdown of invoice concepts, a graphic about the destination of the bill amounts, data about energy consumption by periods, and contact information. We introduce blue capital letters in these figures to name each group. These will be used later for codifying the labels in the following sections. Table 1 summarizes the contents of each group. All the labels and their codes are explained in the Data Records section.

**Table 1:** Groups of labels.

ID	Description	Range	Details
A	Customer who receives the invoice letter	1–6	Table 4
B	Customer data as stated in the contract	1–6	Table 4
C	Marketer data	1-E	Table 5
D	Distributor data	1-D	Table 5
E	Contract information	1–9	Table 6
F	General information about the invoice	1–8	Table 7
G	Customer financial information	1-A	Table 8
I	Energy consumption information	1–3	Table 9
J	Summary of invoice breakdown	1–5	Table 10
K	Detailed invoice breakdown	2-D	Table 11
M	Other billing items, like equipment rental	3, 4	Table 12
N	Taxes	1–8	Table 12

The labels are organized in several groups according to their contents. The group ID is used to code the labels, and the field range stands for the number of labels in the group, coded in hexadecimal. The labels are explained in the corresponding tables in the Data Records section.

One of the main concepts of the bill is energy power, which is the value of the kilowatts (kW) contracted, such as 5.5 KW. This information, together with the price in €/kW, and the number of days, yields the energy power price. Depending on the contracted energy, an access toll increases the amount to be paid. The contracted energy power is the maximum number of kilowatts that can be consumed at once in a household. It establishes the amount of electricity that the customer can consume at a given moment. Roughly speaking, it determines the number of electrical devices that can be connected at the same time. This is a fixed part of the bill and only depends on the number of days and the power rate price. If there is no energy consumption, like in empty houses, this price does not change. The customer may change the energy power, but he may have to pay a fee for this change.

Another important concept is the energy consumed, which is the number of kWh consumed by the customer during the period. The difference between the energy consumed with respect to the earlier period in kWh, and the unit price in €/kWh, yield the price of the energy consumed. The access toll may include time discrimination, which is billed at different rates.

The total amount includes other concepts, such as the equipment rental price or taxes. There are other fields, like the universal supply code–*Código Universal del Punto de Suministro* (CUPS) in Spanish–, the counter and contract number, etc.

### Database design process

The goal of the design process is to create a large dataset of realistic invoices. This process relies on the automatic generation of labels, as explained in the Simulation section below, and a set of document templates. The output is a set of training files in PDF format, and JSON files having the value of labels. Additionally, a set of test documents is generated for evaluation purposes. We choose PDF files by default because most electricity companies issue their invoices in this format. Nevertheless, it is easy to convert them to other formats, such as image files or XML.

Before generating the dataset, we collect several dictionaries with Spanish names and surnames, marketers and distributors registered at the CNMC, villages and streets in Spain, and financial institutions, which are necessary during the simulation process.

On the other hand, we create a set of template documents from real invoices obtained from the main marketers in Spain, i.e., Iberdrola, Endesa, Naturgy, EDP, and Repsol. These are converted to DOCX format to easily edit the contents. Then, we insert the label codes that need to be replaced during the simulation. The fields are annotated between braces, such as {{A1}} or {{DC}}. These codes are composed of two characters, the first one corresponding to a specific group–see Table 1–and the second one to the number of the field in that group. Creating a new template is easy since it requires inserting the codes in a new document only once.

**Fig. 5:**
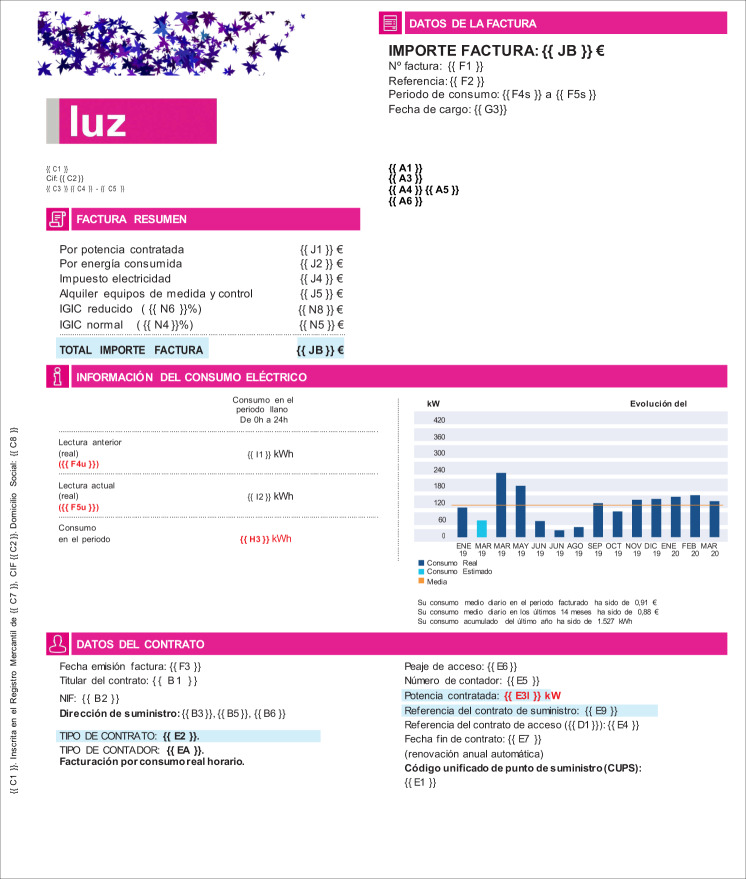
Design of templates. The original invoices are modified by including two-letter codes that stand for the labels. These codes are replaced with simulated data. The first letter of the code stands for the group and, the second one, for the number of the field in that group.

Figure 5 shows an example of a template. These files are used in the pipeline for replacing the codes with simulated data.

**Fig. 6:**
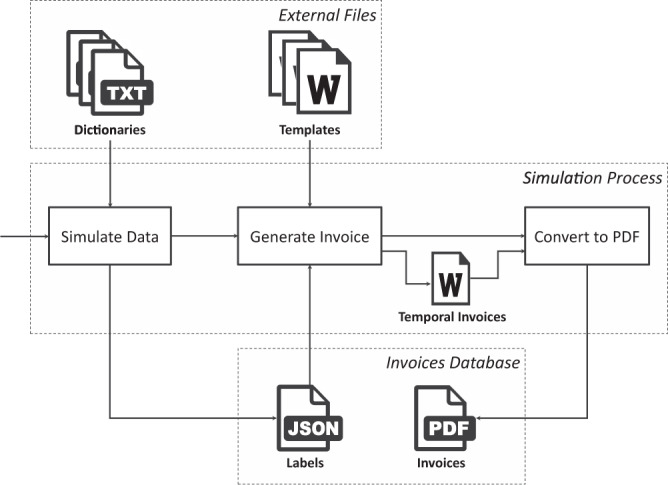
Pipeline for creating the database. The process relies on several dictionaries of customers’ names and surnames, marketers’ and distributors’ information, name of villages and streets, and financial institutions. On the other hand, there are several document templates that are initially configured from real invoices. The pipeline includes three main steps: the first one simulates the contents of the bills and stores the labels in JSON files; the second one fills out the templates using the labels of the previous step; the third step converts the results to PDF format. The output is the database composed of a training and a test directory.

**Fig. 7:**
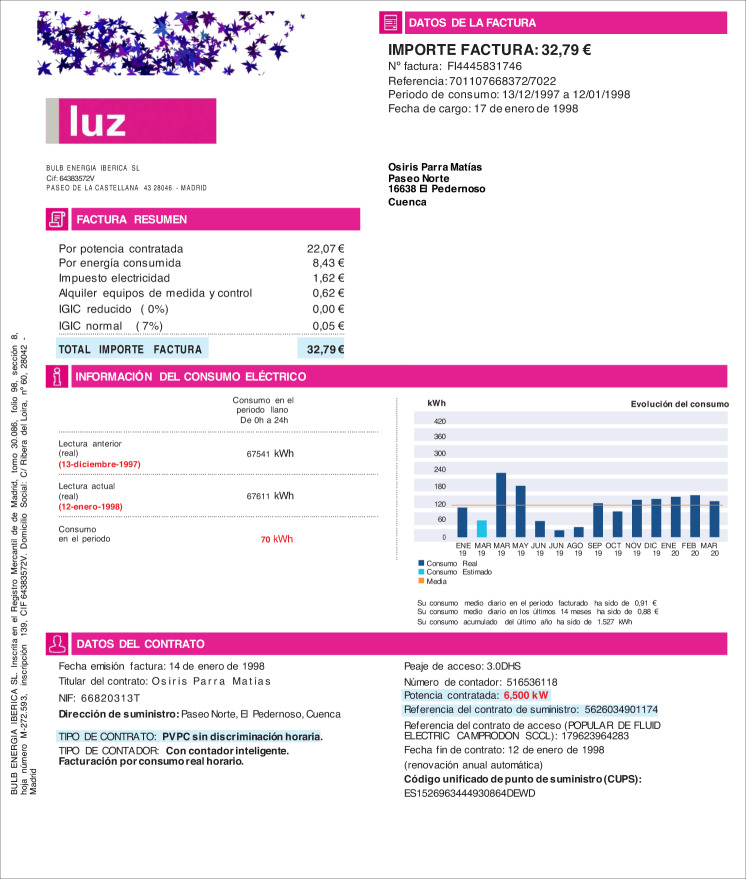
PDF file. Fields in the template are replaced with simulated data, obtaining a PDF file of the invoice.

**Fig. 8:**
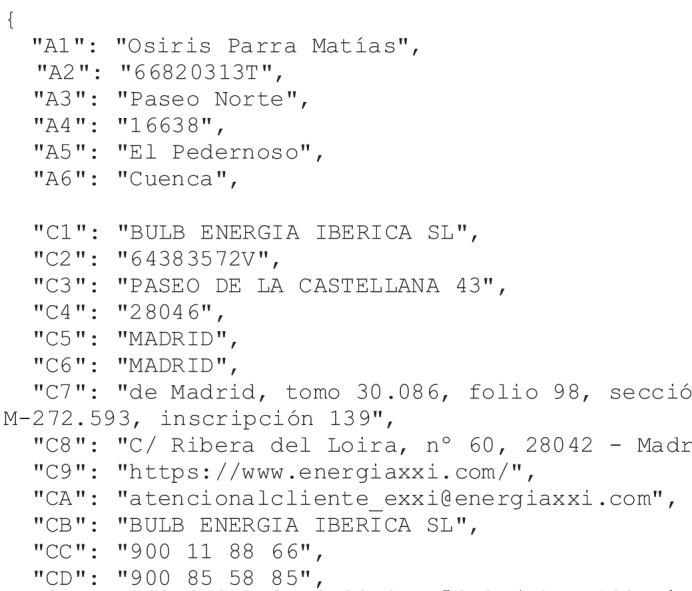
JSON file. Labels of each training sample are written to a JSON file.

The design of the database follows the pipeline shown in Fig. 6. In the first step, we generate random data using dictionaries and several statistics related to the principal amounts of the bill, as explained in the Simulation section. This step creates a JSON file for each invoice. In the next step, the JSON file is used to fill out the document template and generate the final bill in DOCX format. It is in this step that the codes are replaced by the simulated data. In the last step, the document is converted to PDF format. Figures 7, 8 show the result of an invoice and its corresponding labels in the JSON file, respectively.

This process is carried out multiple times for each template to obtain many bills. We create a directory for the template and generate the same number of items in each one. In the training set, we include two PDF files: one for the invoice and another one using annotations around the labels for making it easy to extract the information during the training process. In this case, the label codes are placed at the beginning and end of the corresponding value, such as #A1 Juan Fernández Gutiérrez #A1, with A1 being the code of the customer’s name.

Automatic invoice generation is used in other works^13^. In this case, the authors implement several strategies for generating the contents, such as the use of regular expressions, the connection to a database source, or the generation of random values with given constraints. It also allows generating PDF files with arbitrary layouts and XML files with annotations.

Another work^14^ allows the creation of large datasets of various business documents, such as invoices, payslips, and receipts. Document contents and layouts are randomly generated in PDF, XML, and TIFF formats. The system uses a set of variables and concepts to create a large diversity of samples.

We also generate the contents using regular expressions, external files with tabulated values, and random variables that depend on each field, as in these works. The main difference in our work is that we obtain the data from a simulation process based on national regulations of the electricity market and statistics from the CNMC and REE.

### Simulation of the invoice data

Obtaining a significant number of invoices is difficult due to data protection laws. For this reason, it is more feasible to perform a simulation of the bill amounts. Additionally, a benefit of this approach is that it allows us to obtain ground truth solutions without the need for manual annotations. Most of the bill prices can be calculated from a few basic values, such as the consumed energy or the contracted price per kWh.

Spanish regulations contemplate two alternatives to establish energy prices: the regulated and the free market. In the former, energy prices are set daily by REE in an auction based on the energy offer and demand. In the latter, the marketer and the client negotiate prices between them. Although the amounts vary between both systems, the method for calculating the billing data is the same. In order to simplify the simulation process, we take ideas from both systems depending on the availability of data and the ease of implementation.

The method for estimating prices in the electricity market is regulated in the BOE^15^. We simplify some calculations when they do not contribute to obtaining better results, and we do not include special situations, such as discounts, multiple energy prices depending on the billing period, locale information, or refunds for claims. These situations are difficult to model because there is not enough information available to do the simulation. Nevertheless, these special situations do not affect the rest of the invoice amounts in general.

Tiered energy prices are probably the more interesting information, especially in the regulated market where the energy and contracted power are billed at different rates depending on the time schedule. The most difficult issue here is to simulate these special situations since we do not have statistical information about the distribution of consumption in the three tiers. In the future, we will consider introducing this classification when we have enough data.

These special situations should not affect the design of new machine-learning methods. Introducing this information means including a few more labels, which are a small percentage of the actual number of labels. The methods should work in the same way when trained with these new labels. In the document templates, we removed the parts corresponding to these situations and, in some cases, substituted the contents with capital Xs.

The simulation relies on the following dictionaries:

- Customers’ names and surnames: we obtained a list of 1960 and 455 Spanish names and surnames, respectively. Some of them were compounded, such as *José María* or *De la Cruz*.
- Marketers and distributors: two files have the information of marketer and distributor companies in Spain, obtained from the CNMC. The available marketer data were the name, address, phone, website, and tax identification code, and the distributor data were the name, phone, website, and tax identification code.
- Villages: another file contains the Spanish villages and cities, with their provinces and postal codes, which are used for the customers’ addresses. We use several labels to represent these fields.
- Streets: we use a file to store the name of more than 30 000 streets. The information in this file was combined with the villages to obtain the whole customer’s address.
- Banks: a list of banks registered at the Bank of Spain is stored in another text file. These data were used to complete the customers’ financial information.

Items in these dictionaries were randomly selected using a uniform distribution, so there is an equal probability of picking any value. Customers’ names were also converted to upper letters in 50% of cases. On the other hand, we generated some structured fields, such as the customer’s identity card number, the CUPS, or the contract number, respecting the main structure of the fields and generating part of the contents randomly. Since these labels do not influence any other calculation, we do not check if they correspond to real data. Other fields, like the contracted energy power, or the access toll, were selected from a set of values.

The *start date* of the billing period was calculated randomly between 1990 and 2021. The rest of the dates–*end date*, *issue date*, and *due date*–were calculated considering a one- or two-months period, which is typical in most bills. In some cases, several amounts are calculated using the total number of days, so we do the conversion between the number of months and days.

The simulation selected the format of the dates according to the given template. For this, we added a letter code to date labels, for example, a start date with code #F4l stands for a large format as 12 de enero de 2021, whereas an end date with code #F5s stands for a short format as 12/01/2021; see Table 2. If no code was supplied, then the large format was selected by default. Note that several formats may appear in the same template.

**Table 2:** Date formats.

Label	Format	Example
l	‘%d de %B de %Y’	12 de enero de 2021
s	‘%d/%m/%Y’	12/01/2021
u	‘%d-%B-%Y’	12-enero-2021
p	‘%d.%m.%Y’	12.01.2021

We use several modifiers in the templates to change the format of date labels.

The two main amounts for calculating the bill price are the contracted energy power and the energy consumption. The energy power is the maximum number of kilowatts that cannot be exceeded by all active household appliances at the same time. Currently, its price is fixed at 38.043426 €/kW^16^ per year in the regulated market and is increased by the marketing cost, which is between 0.008219 and 0.010958 €/kW per day, or 3 and 4 €/kW per year. We use a Normal distribution to obtain random values in this range. The CNMC publishes statistical information^17^ about the structure of the national energy system every year. This includes statistics about the number of customers, the distribution of access tolls, the average energy consumption, and some billing data.

The number of customers in the Spanish electricity market–including all types of accounts: residential, commercial, and industrial–was 28 480 765 in 2021, with 99.61% of customers having a low voltage contract (less than 1 kV). Table 3 shows the relation between the types of access tolls, the percentage of customers, and the average price for each toll. The average price of all low voltage tolls is 0.0976 €/kWh. We do not include high voltage contracts because they represent less than 0.4% of the total market.

**Table 3:** Percentage of customers and average prices for each access toll. Source: CNMC^17^.

Access Toll	% Electricity Customers	Average price per day
2.0 A	57.34%	0.1263 €/kWh
2.0 DHA	36.71%	0.0992 €/kWh
2.0 DHS	0.16%	0.0772 €/kWh
2.1 A	1.46%	0.1437 €/kWh
2.1 DHA	1.19%	0.0899 €/kWh
2.1 DHS	0.01%	0.0894 €/kWh
3.0 A	2.68%	0.0633 €/kWh

The contracted power of 94.23% of customers is smaller than 10 kW and, according to a study of the CNMC, the mean contracted power was 4 kW in 2021^17^. We choose a Normal distribution for sampling the values in this range, with a mean of 4 and a standard deviation of 1. The price of the energy power is calculated by multiplying the contracted power by its unit price and the number of billing days. The access toll is obtained from Table 3 following the distribution given by the percentage of customers.

According to REE, the average electricity consumption per month in Spain is 270 kWh. Consumption of 0 kWh is typical in houses that are not inhabited–in this case, the price of the invoice is determined by the contracted power–. In order to simulate these values, we use a Normal distribution with an expected value equal to the average consumption of 270 kWh and a standard deviation of 200, truncating negative values to zero. The unit price is sampled using a Normal distribution based on the average price of the selected access toll in Table 3 and a standard deviation of 10% of the price. This amount can also be expressed per year in some invoices.

The electricity meter rental price is regulated in the BOE^18^ and depends on the type of meter. The prices are among these values: {0.03, 0.15, 0.47, 0.54, 0.72, 0.81, 0.91, 1.11, 1.36, 1.53, 1.71, 2.22, 2.79} €/month. In some bills, the final amount is calculated as a daily price multiplied by the number of days. In our simulation, we pick a random value in this set using a uniform distribution. We use this distribution because we did not find any official information regarding the distribution of electricity meters in Spanish households. We know that most meters are *smart*, but we do not know how many different models there are and what their prices are. This approach is interesting for training machine learning algorithms, as it does not contribute to biasing the algorithms towards certain values.

The electricity tax is regulated and is only applied to the sum of the contracted energy power and the energy consumed. Its value is fixed at 5.1127%. The value-added tax (VAT) is applied to the total amount and its value may depend on the autonomous region. Typical values are in {6.5%, 7%, 10%, 21%}.

The simulation does not check that the calculations are congruent and that physically infeasible situations do not occur. Nevertheless, since we use normal distributions for the main quantities, and most prices are calculated from these quantities, physically infeasible situations are unlikely to occur. We have checked many invoices by hand and have not found inconsistent data.

## Data Records

Each invoice of the training dataset is composed of a document in PDF format, with the bill’s contents, and a JSON file with the value of labels. These labels contain the information necessary to generate the PDF file. Invoices in the test set do not include the JSON files, as this set is used for evaluation purposes.

In this database version, we included nine templates from eight companies. Two pairs of templates are similar because they belong to the same company. We generated 30 000 training documents (5000 documents in each template directory), with their corresponding JSON files, and 45 000 test documents. Three templates in the test set were not included in the training set.

There are 86 different labels, although each template does not necessarily include all of them. Tables 4–12 describe all the labels. Electricity invoices contain many data types, like single-valued fields–such as money, electricity power, alphanumeric codes (invoice number, identity card number, postal or CUPS codes), email, etc.–and multi-valued fields–such as the names and addresses of customers, marketers, and distributors–.

**Table 4:** Customer labels.

Label	Description
A1/B1	Customer’s name
A2/B2	Customer’s identity card number
A3/B3	Customer’s address
A4/B4	Postal code
A5/B5	Customer’s city
A6/B6	Customer’s province

**Table 5:** Marketer and distributor labels.

Label	Description
C1	Marketer’s name
C2	Marketer’s tax identification code
C3	Marketer’s address
C4	Postal code
C5	Marketer’s city
C6	Marketer’s province
C7	Company information in the commercial register
C8	Address of the company administration
C9	Marketer’s website
CA	Marketer’s email
CB	Short company name
CC	Marketer’s contact telephone
CD	Customer’s support telephone
CE	Marketer’s telephone for claims
D1	Distributor’s name
D2	Distributor’s tax identification code
D9	Distributor’s website
DC	Public service phone
DD	Telephone for breakdowns

**Table 6:** Contract labels.

Label	Description
E1	Universal Supply Point Code or *Código Universal del Punto de Suministro* (CUPS) in Spanish
E2	Contract type or rate
E3	Contracted electricity power
E4	Contract number
E5	Electricity meter number
E6	Access toll
E7	Contract end date
E8	Code for the National Classification of Economic Activities, or *Clasificación Nacional de Actividades Económicas* (CNAE) in Spanish
E9	Reference supply contract

**Table 7:** Billing information labels.

Label	Description
F1	Invoice number
F2	Invoice reference
F3	Invoice release date
F4	Start billing date
F5	End billing date
F6	Total number of billing days
F7	Days per year
F8	Number of months

**Table 8:** Payment information labels.

Label	Description
G1	Payment method
G2	IBAN (International Bank Account Number)
G3	Payment date
G4	A sequence of numbers that identify the payment operation
G5	A sequence of numbers that identify the payment operation with G4
G6	Bank code
G7	Office code
G8	Control digits
G9	Bank account number
GA	Bank name

**Table 9:** Energy consumption labels.

Label	Description
I1	Energy consumption in kWh at the previous period
I2	Energy consumption in kWh at the current period
I3	Number of kWh consumed in the period. It is the difference between I1 and I2

**Table 10:** Price labels.

Label	Description
J1	Contracted electricity power price
J2	Energy consumed price
J3	Subtotal 1. It is the sum of J1 and J2
J4	Subtotal 2. Price without taxes. It is the sum of J3 and N3
J5	Total price of the invoice

**Table 11:** Invoice breakdown labels organized by the contracted energy power (Power) and the energy consumed (Energy).

	Label	Description
Power	K2	Access toll rate (€/kW). Invoices may include the toll rate as €/kW per year (K2) or €/kW per day (K2d)
	K3	Access toll price (€)
	K4	Marketer cost rate (€/kW)
	K5	Marketer cost price (€)
	K6	Contracted power rate obtained as the sum of K2 and K4
Energy	K9	Access toll energy rate (€/kWh)
	KA	Access toll energy price (€)
	KB	Energy cost rate (€/kWh)
	KC	Energy cost price (€)
	KD	Energy rate obtained as the sum of K9 and KB

**Table 12:** Tax labels.

Label	Description
M3	Equipment rental price (daily price)
M4	Equipment rental price
N1	Electricity tax rate
N2	Electricity tax price
N3	Sum of M4 and N2
N4	Normal tax rate
N5	Reduced tax price
N6	Reduced tax rate
N7	Sum of N2 and KF
N8	Tax price

Table 4 shows the labels corresponding to the customer information. We differentiate between the customer who receives the invoice letter (group A) and the customer who signed the contract (group B). There are six labels, which include the customer’s name, identity card, and whole address.

The marketer and distributor labels are detailed in Table 5. These include the postal address, contact information, and the company website. In the case of the distributor, we consider its name, website, tax identification code, and several telephones.

Labels related to the contract are given in Table 6. These include the CUPS, the contract type, the contracted electricity power, the meter, the access toll, etc.

Table 7 shows the labels of the billing information. These include the invoice number and billing dates. Fields related to the payment method are detailed in Table 8, with the customer’s bank account and payment date.

The energy consumption labels are given in Table 9. Some companies only include the total energy consumption in the current period. Other marketers include the energy consumption in the current and previous periods, as given by the electricity meter, and the final consumption is calculated as the difference between these two values.

Table 10 shows the invoice breakdown labels. Although the organization of this information is different for each marketer, it typically preserves a similar structure. The electricity power, the energy consumed, and the taxes are present in all invoices, however, other concepts, like the equipment rental price, or subtotals are not always included.

Table 11 enumerates the labels of the contracted energy power and consumed energy prices and rates. Finally, Table 12 contains the labels related to different tax rates and prices.

The IDSEM dataset is available through Figshare^19^ and Zenodo^20^. In the latter, there is a unique compressed file that contains two sub-directories, one for the training set and another for the test set. The size of this file is 30.9 GB. In the Figshare repository, there are two zip files with the training and test sets, with 13 GB and 17 GB, respectively. Additionally, we have included separate files with a subset of the directories in each set, so it can be downloaded by parts. There is also a reduced version of the dataset with 100 invoices per directory, which is interesting for users who want to preview the content of the dataset before downloading it.

## Technical Validation

**Fig. 9:**
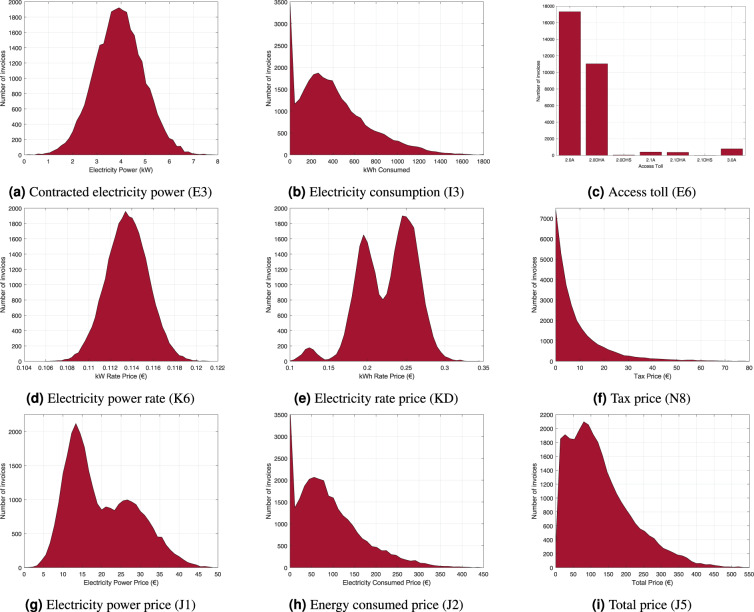
These figures show the distributions of several labels. From the JSON files in the training directory, we calculated the histograms of the most important amounts in the bills.

In this section, we analyze the technical quality of the database. Figure 9 shows graphs with the distribution of several labels. We process the 30 000 JSON files in the training directory and obtain the histograms of the main amounts. Approximately equivalent results are obtained for the test set. The statistics obtained from the training data confirm the values used in our simulation.

Figure 9a shows the graph of the contracted electricity power, which follows a Normal distribution with a mean of 3.99 and a standard deviation of 1.0049. Figure 9b is the histogram of the electricity consumed in kWh. We observe a large concentration in 0 kWh due to many households with no consumption, representing a 17% of the total. There is no official data that confirms this percentage, but we know that the number of empty houses in Spain is around 13.7%, as reported by the Instituto Nacional de Estadística (INE).

Additionally, we may think of houses for rent that are not occupied, waiting for a new renter, or second homes that are typically empty during most months of the year. Although 17% may seem reasonable for houses with no electricity consumption, what is important for machine learning algorithms is that this situation is represented in the dataset with enough documents. We observe another peak around 270 kWh, which corresponds to the mean reported by REE.

Figure 9c shows the number of contracts by access toll. We observe that this graphic follows the distribution given in Table 3 obtained from the CNMC. The most frequent tolls are 2.0 A and 2.1 DHA with 57.78% and 36.81%, respectively (94.59% in total).

The electricity power rate price follows a Gaussian distribution; see Fig. 9d. Its mean value is 0.1137 €/day, which corresponds to the daily cost of the electric power price and marketing costs; see the Simulation section above. Its standard deviation is 0.00193.

The electricity rate price–Fig. 9e–depends on the access toll and its distribution agrees with the information given in Table 3. The three peaks account for the price of the most frequent tolls, i.e., 2.0 A, 2.0 DHA, and 3.0 A, which are 0.2526€, 0.1984€, and 0.1266€, respectively. The magnitude of these peaks corresponds to the percentage of customers with these tolls.

The graph of the electric power price is shown in Fig. 9g. This amount is determined by the access toll, the number of days, and the electric power rate; see Fig. 9d. The mean value is 20.4€ and the standard deviation is 8.69€.

The electricity price–Fig. 9h–depends on the energy consumed and the electricity rate price; therefore, it is a combination of the graphs in Fig. 9b,e. The minimum value is 0€ and the maximum is 557.77€. The mean value is 95.25€ and the standard deviation is 76.15€. The distribution of taxes is given in Fig. 9f, with a mean of 9.61€ and a standard deviation of 11.29 €.

Finally, the total price distribution is given in Fig. 9i. The minimum, in this case, is 4.34€, the maximum is 664.49€, the mean is 132.99€ and the standard deviation is 90.89€. Note that, in this case, the values are never zero because the electric power price is always bigger than zero.

In order to ensure that the generated documents were correct, we conducted a manual validation. Before generating the final training and test sets, we created a reduced version of the database with one hundred documents for each template. Then, we randomly selected ten invoices from each directory and verified that the data were correct.

We checked that the dates were in the correct ranges and the customer, marketer, and distributor information were copied in the correct places. We verified that prices and billing amounts were correctly calculated and paid special attention to rounding errors.

In the validation process, we also checked the format of the bills, comparing the layout, text fonts, and margins with the original templates. On the other hand, we also checked the contents of the JSON files, looking at the correspondence between the labels and their values.

## Usage Notes

There are several software packages that are suitable for analyzing this database. One example is MATLAB, which contains many libraries that implement machine learning algorithms and many useful functions for dealing with data-intensive applications. Another alternative is to use packages from the Python programming language. The most important libraries are Keras^21^ and PyTorch^22^. These are focused on neural networks and contain many recent deep learning algorithms that are ideal for testing the dataset. The scikit-learn^23^ library contains many traditional machine learning algorithms and many functionalities for manipulating large datasets and evaluating the results.

In the case of Python, several packages allow for managing PDF and JSON files. For instance, the pdf2text package allows converting a PDF file to text format. This is interesting, e.g., for working with natural language processing pipelines. In this case, the main steps for processing the data are usually tokenization, part-of-speech tagging, dependency parsing, lemmatization, stemming, stopword removal, named entity recognition (NER), etc. This approach produces a sequence of text data that can be easily processed with recurrent neural networks.

Another approach may treat documents as images. This is interesting for applying computer vision techniques, especially those based on convolutional neural networks. The pdf2image package can convert PDF files to several image formats. In this case, it would be interesting to apply an optical character recognition (OCR) system in order to obtain the text from images. A standard application for this purpose is tesseract.

The scikit-learn^23^ library contains algorithms for evaluating the performance of methods. Since this database is intended for the extraction and analysis of information, it is reasonable to use standard performance metrics, such as Precision, Recall, and F1-score. The accuracy obtained for chunks of words, like names and addresses, can be done with metrics that compare the similarity of strings, such as the Levenshtein distance.

Although most electricity companies issue their bills in PDF format, we may assume that documents have been scanned from a smartphone. In this case, the database must have images in PNG or JPEG format. Users can make this conversion using the pdftoppm program. However, simulating the effects of scanning is not an easy task and could take a lot of work–this can be addressed in future versions of the database–. This would introduce new challenges for machine learning algorithms, involving techniques for manipulating the images.

## Data Availability

The code used to generate the dataset is released under the BSD license and is available at Zenodo^24^. The repository has a directory with invoice templates in DOCX format and another directory with the dictionaries described above. The code of the pipeline was implemented in Python 3. For the first step, we used standard functions for simulating the data, based on the random, time, math, os, and string libraries. We also used the json library for working with JSON files. We used the docxtpl library to replace fields in the DOCX templates with the JSON data in the second step. Internally, this library relies on two packages: python-docx for reading, writing, and creating documents; and jinja2 for managing tags in the template. The DocxTemplate function allows replacing the text between braces with its corresponding value in the JSON file. The result is written in a DOCX file. Finally, this file is converted to PDF using the docx2pdf library. The program can be executed from the command line as python3 main.py. In order to generate the final version of the database, the program was executed in an Intel Core i9 CPU with 14 cores and 32.0 GB RAM, under Windows 10. The size of the database is approximately 30 GB in the disk. Each invoice may have between 2 and 4 pages, and the size of each PDF file on disk is between 160 KB and 1.68 MB. The size of JSON files is between 3 KB and 4 KB. The database contains 135 000 files with six and nine directories in the training and test sets, respectively. The test set has 45 000 invoices in PDF format and the training set has 90 000 files: 30 000 invoices in PDF format; 30 000 annotated invoices in PDF format; and 30 000 JSON files with the value of labels. In the non-distributable database, we have also included annotated invoices and labels for the test set, which amounts to 45 000 PDF and 45 000 JSON files, respectively. Therefore, the total number of files in this version is 225 000 files, which occupy 49.7 GB of disk. Regarding the execution time for the generation of the database, we calculated the average time of each step in the pipeline for each invoice; see Fig. 6. This average time was calculated after eight runs with 500 invoices. The results in seconds per bill are as follows: • Simulation process: 0.036 s • Generation of the invoice file in DOCX format: 0.544 s • Conversion from DOCX to PDF: 2.053 s Therefore, the average time for creating one invoice was 2.633 seconds. The slowest step was the generation of PDF files (77.98% of the total time), followed by the creation of invoices (20.67%), and, finally, the simulation process (1.35%). The total run time was approximately 197 475 seconds, or 54.85 hours. The process was executed in several batches.
